# Increased fragility fracture risk in Korean women who snore: a 10-year population-based prospective cohort study

**DOI:** 10.1186/s12891-017-1587-0

**Published:** 2017-05-31

**Authors:** Soo Beom Choi, Il Suk Lyu, Wanhyung Lee, Deok Won Kim

**Affiliations:** 1 Department of Medical Engineering, Yonsei University College of Medicine, CPO Box 8044, Seoul, Korea; 2 Graduate Program in Biomedical Engineering, Yonsei University, Seoul, Korea; 3 Department of Medicine, Yonsei University College of Medicine, Seoul, Korea; 4 Graduate School of Public Health, Yonsei University College of Medicine, Seoul, Korea

**Keywords:** Cohort study, Snoring, Fracture, Osteoporosis, Pre-screening

## Abstract

**Background:**

Snoring is frequently associated with obstructive sleep apnea (OSA). Previous studies have shown that bone mineral density was significantly lower in patients with OSA than in controls; however, these studies did not focus on fractures. Fragility fractures can lead to long-term disabilities and a decrease in quality of life. The present study aimed to investigate the association between snoring and fragility fractures.

**Methods:**

This study included 2969 men and 3220 women aged 40 years and older from the Ansung and Ansan cohort studies in Korea. During a 10-year follow-up period, 129 and 273 fracture cases were reported in men and women, respectively.

**Results:**

Severe snoring (6–7 nights per week or sleep disturbance by snoring in the next room) was a statistically significant risk factor for fracture (*p* = 0.006, hazard ratio 1.68, 95% confidence interval 1.16–2.43) after adjusting for covariates related to fragility fracture in women. However, both snoring and severe snoring groups did not show significant associations with the fracture risk in men.

**Conclusions:**

Thus, information on the frequency of snoring in women may improve the accuracy of fragility fracture risk prediction, which can help in deciding whether intervention or treatment is necessary.

**Electronic supplementary material:**

The online version of this article (doi:10.1186/s12891-017-1587-0) contains supplementary material, which is available to authorized users.

## Background

Fragility fractures are a major and increasingly common cause of morbidity, and they pose a considerable burden on healthcare systems [1]. Moreover, most osteoporosis-related fractures can lead to significant long-term disabilities and a decrease in the quality of life [2, 3]. Screening for low bone mineral density (BMD) using dual-energy X-ray absorptiometry (DXA) is an accepted strategy to identify people with an elevated risk of fragility fracture. However, mass screening with DXA in the general population, without pre-screening for high-risk individuals, is generally not recommended [4]. Moreover, people who have not experienced fractures may not recognize the necessity of having their BMD measured; therefore, people with osteoporosis may not realize that their BMD is low before fragility fractures actually occur. Thus, it is important to identify risk factors for fragility fractures that can be easily assessed without special examination.

Obstructive sleep apnea (OSA) syndrome is a common disorder characterized by recurring episodes of apnea or hypopnea due to total or partial pharyngeal collapse during sleep [5]. Previous studies have reported on the risk of osteoporosis in patients with OSA. A previous retrospective study of 66 chronic obstructive pulmonary disease (COPD) patients found that lumbar spine BMD was significantly lower in COPD patients with OSA than in COPD patients without OSA [6]. Additionally, a 6-year retrospective cohort study of 1377 OSA patients and 20,655 matched controls found that the risk of osteoporosis was 2.52-fold higher in the OSA patients than in the matched controls [7]. Moreover, a 10-year retrospective cohort study of 846 OSA patients and a control group of 89,380 people with no sleep disorders found that OSA patients had an adjusted hazard ratio of 2.98 for osteoporosis when compared with the control group [8]. Furthermore, a cross-sectional study of 26 male OSA patients and 21 male participants without OSA showed that the spine and femoral neck BMDs were significantly lower in the OSA patients than in the participants without OSA [9].

Snoring is frequently associated with OSA, and patients who initially have only primary snoring may be at risk for the future development of OSA with increasing age or weight gain [10]. However, the above-mentioned previous studies focused on BMD and not fragility fractures. Moreover, studies on the association between snoring and fragility fracture risk are rare. Therefore, the objective of the present study was to investigate the association between snoring and fragility fractures in elderly people using data obtained in a cohort study performed in Korea.

## Methods

### Study population

**Fig. 1:**
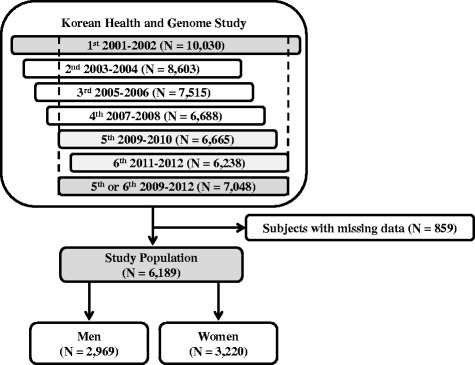
Flow chart of participants for the Ansung and Ansan cohort studies with subject exclusion

Two communities were selected for the Korean Health and Genome Study (KHGS) in 2001; the Ansung and Ansan cohorts represented rural and urban communities, respectively. This is an ongoing prospective study involving a biennial examination. Detailed information on the study design and procedures has been published previously [11]. At each visit, informed written consent was obtained from all participants. Initial data were obtained from the 10,030 subjects who participated in the Ansung and Ansan cohort studies. The fifth and sixth follow-up examinations (2009–12) collected data related to fractures. Among the 10,030 subjects, 2982 did not participate in the fifth and sixth follow-up examinations and were thus excluded. Among the remaining 7048 subjects, 859 subject had missing data. These subjects were excluded from this study. Therefore, 2969 men and 3220 women were finally included in this study (Fig. 1). The institutional review board of the Yonsei University Health System approved the protocol of this study (No. 4-2016-0572).

### Selection of fracture events

Fracture events were recorded using a standardized self-administered questionnaire. In this study, we only included fracture events that occurred when the participants fell while walking or while performing activities of daily living. Fractures clearly caused by high-trauma events, such as motor vehicle accidents, violence, and falls from more than the standing height of the individual, were excluded [12]. In addition, we considered fracture events from all sites.

### Clinical risk factors

Information on the presence of snoring was obtained using a questionnaire administered face to face. One of the questions asked was “Have you ever been told that you snored? If so, how many nights per week? The response options were “rarely,” “1–3 times a week,” “4–5 times a week,” and “6–7 times a week.” Another question asked was “Have you ever been told that your snoring disturbed a person’s sleep in next room?” The incidences of fractures in women were 7.4% (*n* = 103) among those who did not snore, 8.4% (*n* = 108) among those who rarely snored, 9.5% (*n* = 15) among those who snored 1–3 times a week, 8.6% (*n* = 8) among those who snored 4–5 times a week, and 13.7% (*n* = 39) among those who snored 6–7 times a week. Based on the statistical analysis for optimal criterion of snoring frequency, snoring frequency was classified into the following 3 groups: non-snoring, snoring (1–5 nights per week), and severe snoring (6–7 nights per week or sleep disturbance by snoring in the next room). In this study, the covariates for fracture included age, weight, height, body mass index, waist circumference, hip circumference, regular exercise, alcohol consumption, lifetime smoker, history of previous fracture, family history of osteoporosis or fracture, degenerative arthritis, rheumatoid arthritis, hypertension, diabetes mellitus, medications for osteoporosis, years since menopause, estrogen therapy, and BMD. Regular exercise was defined as engaging in any of a variety of activities for the purpose of exercise at least once per week. The smoking and alcohol consumption variables were smoking status (never, former, or current) and alcohol consumption status (never, former, or current), respectively. The number of years since menopause was defined as “0” for premenopausal subjects and was the actual value for postmenopausal subjects.

Quantitative ultrasound (qUS) measurements of BMD at the radius and tibia were performed using the Omnisense 7000 device (Sunlight Medical, Ltd., Rehovot, Israel) with a handheld probe specifically designed for the measurement of the axial speed of sound (SoS, m/s) along the surface of bone [12]. In addition, T and Z scores were calculated in both the radius and tibia.

### Statistical analysis

The characteristics of the study participants are reported as mean ± standard deviation (SD) for continuous variables and as number (%) for categorical variables. In order to evaluate the association between each of these variables and the fragility fracture risk, analysis of variance (ANOVA) was performed for each continuous variable and the chi-square test was performed for each categorical variable.

Cox regression was performed to investigate whether snoring was associated with an increase in the risk of fragility fractures. Among the Cox regression analysis results, hazard ratios and 95% confidence intervals were reported. The survival time was defined as the time interval between the first examinations (2001) and the first fragility fracture since the first examinations. Non-fracture cases were censored at the time of the sixth follow-up examinations (2011). First, crude univariate Cox regression was performed without controlling for any covariate. Second, Cox regression analysis was performed after controlling for age, weight, height, waist circumference, hip circumference, regular exercise, alcohol consumption, lifetime smoker, history of previous fracture, family history of osteoporosis or fracture, degenerative arthritis, rheumatoid arthritis, hypertension, diabetes mellitus, years since menopause, estrogen therapy, and qUS measurements at the radius and tibia. Third, a Cox regression model with backward stepwise elimination was used to restrict adjustment for confounding factors with a threshold of *p* = 0.1. The Cox regression model proportionality of hazard assumption was verified using the log-minus-log method (LML) and time-dependent coefficients.

Finally, Kaplan-Meier analysis was performed in the non-snoring, snoring, and severe snoring groups, and the log-rank test was performed to check for statistically significant differences among these 3 groups. All statistical analyses were two-sided and were performed using SPSS 23.0 (IBM Corp., Armonk, NY). A *p*-value <0.05 was considered statistically significant.

## Results

### Clinical characteristics of fractures

The mean (SD) survival time of the study population was 9.5 (1.3) years for women. During the follow-up period, 273 women (8.5% of all women) experienced fractures. Among these 273 fracture cases, 52 (19.0% of all fracture cases) involved the hip, 14 (5.1%) vertebrae, 33 (12.1%) wrist, 3 (1.1%) coccyx, 16 (5.9%) humerus, 17 (6.2%) forearm, 25 (9.2%) rib, 80 (29.3%) lower limb, 1 (0.4%) clavicle, and 32 (11.7%) other locations. The mean (SD) survival time of the fracture cases was 6.6 (2.4) years. The mean (SD) survival time of the study population was 9.6 (1.1) years for men. During the follow-up period, 129 men (4.3% of all men) experienced fractures (Additional file 1: Table S1).

The results of ANOVA and the chi-square test suggested that age, weight, height, body mass index, waist circumference, hip circumference, degenerative arthritis, rheumatoid arthritis, hypertension, diabetes mellitus, years since menopause, SoS at the radius, T and Z scores of the radius, SoS at the tibia, and T and Z scores of the tibia were significantly different between the non-snoring, snoring, and severe snoring groups in women (Table 1).

**Table 1:** Demographic and clinical characteristics of the female participants in the snoring groups (*n* = 3220)

	Non-snoring (*n* = 1398)	Snoring (1–5 nights/week) (*n* = 1493)	Severe snoring (6–7 nights/week) (*n* = 329)	*p*-value
Survival time (years)	9.6 ± 1.1	9.5 ± 1.4	9.3 ± 1.6	0.003*
Age (years)	51.3 ± 8.9	52.6 ± 8.6	55.7 ± 8.2	<0.001*
Weight (kg)	57.1 ± 7.8	59.8 ± 8.2	62.5 ± 8.6	<0.001*
Height (cm)	153.9 ± 5.6	154.1 ± 5.5	153.5 ± 5.5	<0.001*
Body mass index (kg/m^2^)	24.1 ± 3.0	25.2 ± 3.1	26.5 ± 3.2	<0.001*
Waist circumference (cm)	79.8 ± 9.2	82.3 ± 9.5	86.6 ± 9.2	<0.001*
Hip circumference (cm)	92.4 ± 5.7	93.9 ± 5.8	96.2 ± 6.2	<0.001*
Regular exercise, *n* (%)	325 (23.3)	366 (24.5)	69 (21.0)	0.359
Alcohol consumption, *n* (%)				0.314
Never	1012 (72.4)	1052 (70.5)	221 (67.2)	
Past	32 (2.3)	40 (2.7)	12 (3.7)	
Current	354 (25.3)	401 (26.9)	96 (29.2)	
Lifetime smoker, *n* (%)				0.814
Never	1342 (96.0)	1438 (96.3)	312 (94.8)	
Past	29 (2.1)	29 (1.9)	9 (2.7)	
Current	27 (1.9)	26 (1.7)	8 (2.4)	
History of previous fracture, *n* (%)	31 (2.2)	42 (2.8)	15 (4.6)	0.062
Family history of osteoporosis or fracture, *n* (%)	78 (5.6)	79 (5.3)	18 (5.5)	0.943
Degenerative arthritis, *n* (%)	187 (13.4)	279 (18.7)	94 (28.6)	<0.001*
Rheumatoid arthritis, *n* (%)	98 (7.0)	118 (7.9)	37 (11.3)	0.037*
Hypertension, *n* (%)	172 (12.3)	289 (19.4)	89 (27.1)	<0.001*
Diabetes mellitus, *n* (%)	60 (4.3)	86 (5.8)	35 (10.6)	<0.001*
Medications for osteoporosis, *n* (%)	62 (4.4)	87 (5.8)	17 (5.2)	0.239
Years since menopause (years)	6.5 ± 8.5	7.2 ± 8.5	9.7 ± 8.8	<0.001*
Current estrogen therapy, *n* (%)	55 (3.9)	45 (3.0)	15 (4.6)	0.245
Speed of sound of radius (m/s)	4212.2 ± 185.5	4187.6 ± 186.1	4136.6 ± 187.4	<0.001*
T-score of radius	0.3 ± 1.6	0.1 ± 1.6	−0.3 ± 1.6	<0.001*
Z-score of radius	1.1 ± 1.4	0.9 ± 1.4	0.8 ± 1.4	<0.001*
Speed of sound of tibia (m/s)	3867.6 ± 164.3	3854.3 ± 161.9	3822.2 ± 171.3	<0.001*
T-score of tibia	−0.8 ± 1.5	−0.9 ± 1.5	−1.2 ± 1.6	<0.001*
Z-score of tibia	−0.1 ± 1.3	−0.1 ± 1.3	−0.2 ± 1.5	<0.001*
Fracture, *n* (%)	103 (7.4)	124 (8.3)	46 (14.0)	0.001*

Data are presented as mean ± standard deviation or number of participants (%)

The *p*-values of continuous and binary variables were calculated using analysis of variance (ANOVA) and the chi-square test, respectively

*p < 0.05

### Hazard ratios for fracture

When the non-snoring group was used as the reference group, the snoring and severe snoring groups in women had hazard ratios of 1.14 and 1.97, respectively, in the crude Cox regression model (Table 2). After adjusting for all covariates, the fracture risk was 1.68-fold higher in the severe snoring group than in the non-snoring group (model 2). Moreover, the Cox regression model (model 3) with backward stepwise elimination showed that the fracture risk was 1.68-fold higher in the severe snoring group than in the non-snoring group. However, the snoring group did not show a significant association with the fracture risk in all the models. In both models 2 and 3, severe snoring, height, waist circumference, hip circumference, family history of osteoporosis or fracture, rheumatoid arthritis, and medications for osteoporosis were selected as risk factors for fractures in women. However, both the snoring and severe snoring groups did not show significant associations with the fracture risk in men (Additional file 1: Table S2).

**Table 2:** Crude and adjusted hazard ratios with 95% confidence intervals for fractures among the female participants in Cox regression analyses

	Fracture		Model 1		Model 2		Model 3	
	No (*n* = 2947)	Yes (*n* = 273)	*p*-value	HR (95% CI)	*p*-value	HR (95% CI)	*p*-value	HR (95% CI)
Survival time (years)	9.8 ± 0.6	6.6 ± 2.4						
Snoring, *n* (%)								
Non-snoring	1295 (43.9)	103 (37.7)	Re﻿ference		Reference		Reference	
Snoring	1369 (46.5)	124 (45.4)	0.320	1.142 (0.879–1.483)	0.495	1.097 (0.841–1.432)	0.506	1.094 (0.840–1.424)
Severe snoring	283 (9.6)	46 (16.9)	<0.001*	1.969 (1.391–2.787)	0.006*	1.682 (1.164–2.430)	0.005*	1.680 (1.168–2.416)
Age (years)	52.2 ± 8.8	54.0 ± 8.5			0.178	1.019 (0.991–1.048)	0.007*	1.023 (1.006–1.039)
Weight (kg)	58.9 ± 8.2	59.1 ± 8.3			0.956	0.999 (0.964–1.035)		
Height (cm)	154.0 ± 5.5	153.1 ± 5.5			0.014*	0.966 (0.939–0.993)	0.004*	0.965 (0.941–0.988)
Body mass index (kg/m^2^)	24.8 ± 3.2	25.2 ± 3.2						
Waist circumference (cm)	81.6 ± 9.6	82.1 ± 9.5			0.039*	0.978 (0.957–0.999)	0.006*	0.977 (0.960–0.993)
Hip circumference (cm)	93.4 ± 5.9	94.2 ± 5.7			0.014*	1.047 (1.009–1.086)	<0.001*	1.048 (1.022–1.076)
Regular exercise, *n* (%)	693 (23.5)	67 (24.5)			0.776	1.043 (0.780–1.394)		
Alcohol consumption, *n* (%)								
Never	2088 (70.9)	197 (72.2)			Reference			
Former	74 (2.5)	10 (3.7)			0.375	1.336 (0.704–2.536)		
Current	785 (26.6)	66 (24.2)			0.615	0.929 (0.697–1.238)		
Lifetime smoker, *n* (%)								
Never	2832 (96.1)	260 (95.2)			Reference			
Former	61 (2.1)	6 (2.2)			0.995	0.998 (0.441–2.256)		
Current	54 (1.8)	7 (2.6)			0.423	1.365 (0.638–2.921)		
History of previous fracture, *n* (%)	80 (2.7)	8 (2.9)			0.803	0.913 (0.448–1.862)		
Family history of osteoporosis or fracture, *n* (%)	154 (5.2)	21 (7.7)			0.029*	1.658 (1.052–2.612)	0.028*	1.658 (1.055–2.605)
Degenerative arthritis, *n* (%)	504 (17.1)	56 (20.5)			0.764	0.953 (0.694–1.308)		
Rheumatoid arthritis, *n* (%)	219 (7.4)	34 (12.5)			0.020*	1.563 (1.073–2.278)	0.019*	1.552 (1.076–2.238)
Hypertension, *n* (%)	499 (16.9)	51 (18.7)			0.847	0.968 (0.698–1.342)		
Diabetes mellitus, *n* (%)	165 (5.6)	16 (5.9)			0.860	0.955 (0.569–1.601)		
Medications for osteoporosis, *n* (%)	141 (4.8)	25 (9.2)			0.004*	1.877 (1.225–2.876)	0.004*	1.856 (1.221–2.819)
Years since menopause (years)	7.0 ± 8.6	8.4 ± 8.7			0.821	0.997 (0.973–1.022)		
Estrogen therapy, *n* (%)	104 (3.5)	11 (4.0)			0.771	0.912 (0.492–1.690)		
Speed of sound in radius (m/s)	4195.9 ± 187.3	4162.4 ± 184.5			0.318	1.000 (0.999–1.000)		
Speed of sound in tibia (m/s)	3859.1 ± 164.6	3832.1 ± 160.9			0.627	1.000 (0.999–1.001)		

Data are presented as mean ± standard deviation or number of participants (%)

Crude univariate Cox regression model 1 did not control for any covariate

Cox regression model 2 controlled for age, weight, height, waist circumference, hip circumference, regular exercise, alcohol consumption, lifetime smoker, history of fracture, family history of osteoporosis or fracture, degenerative arthritis, rheumatoid arthritis, hypertension, diabetes mellitus, years since menopause, estrogen therapy, and quantitative ultrasound measurements at the radius and tibia

Cox regression model 3 with backward stepwise elimination involved a threshold of *p* = 0.1

*Abbreviations*: *HR* hazard ratio, *CI* confidence interval

*p < 0.05

### Kaplan-Meier survival analysis and log-rank test

**Fig. 2:**
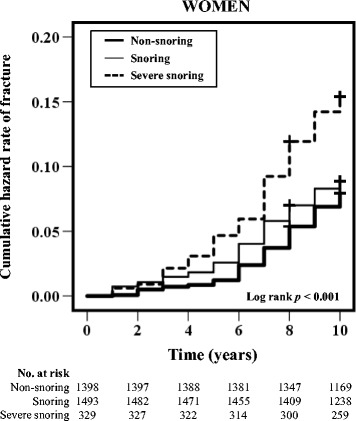
The cumulative hazard rates of fractures and the log rank values for the non-snoring, snoring, and severe snoring groups in women

We performed Kaplan-Meier analyses and log-rank tests to evaluate the association between snoring and fracture risk in women (Fig. 2). The log-rank test results indicated that the fracture risk was significantly higher in the severe snoring group than in the non-snoring group (*p* < 0.001).

## Discussion

In this study, female participants with severe snoring had a statistically significant increase in the fracture risk, even after adjusting for covariates. To the best of our knowledge, the present study is the first to investigate the association between snoring and fragility fractures using data from a 10-year cohort study. Information about snoring can easily be obtained with history taking, and it does not require medical examinations, such as DXA. Thus, we expect that collecting information about snoring may be an inexpensive and simple approach to help increase the accuracy of the prediction of fragility fracture events. Furthermore, if future studies identify the exact mechanism by which snoring increases the fragility fracture risk, treatment of snoring may become an accepted approach to reduce the fragility fracture risk.

This study showed that snoring was significantly associated with fragility fractures in women aged 40 years and older. Although few if any previous studies have specifically investigated the association between snoring and fragility fractures, several previous studies have analyzed the osteoporosis risk in OSA patients [6–9]. Snoring has been reported to be frequently associated with OSA [10]. Moreover, Jennum et al. demonstrated that snoring was associated with the respiratory distress index, which was calculated as the number of apnea and hypopnea episodes lasting longer than 10 s per hour [13]. However, the authors mentioned that self-reported snoring alone was not sensitive enough to identify individuals with sleep apnea [13]. The repetitive hypoxia and subsequent reoxygenation that occur in OSA may lead to oxidative stress, which may result in acidosis because of insufficient vascular perfusion [7]. Furthermore, hypoxia may inhibit the proliferation of osteoblasts and promote proliferation of osteoclasts, resulting in osteoporosis [7, 14]. A decrease in BMD caused by the above-mentioned mechanisms may increase the fragility fracture risk in the elderly.

Suzuki et al. investigated the association between sleep disturbance and hip fracture risk, which may provide more insights into the association between snoring and an increase in the fracture risk [15]. In that study, the authors followed participants for 6 months and reported that there was a significant association between sleep disturbance and hip fracture risk (odds ratio 2.60, 95% confidence interval 1.31–5.17) [15]. Sleep disturbance may increase the fracture risk in the elderly by decreasing the alertness level during the daytime and thus increasing the risk of falling [15]. However, further studies are needed to investigate the exact association between snoring and fragility fracture risk.

Zhang et al. demonstrated the associations between insomnia and falls using cross-sectional data (954 subjects) in Boston, Massachusetts [16]. Adults with insomnia had a 32% increased likelihood of falls after adjustment for multiple covariates, but insomnia was not associated with recurrent falls or fractures. The authors also assessed snoring and falls; however, there was no significant difference. The study for insomnia and falls involved only 2 years of follow-up data. A longer follow-up duration is required for fractures. Moreover, Upala et al. performed a systematic review and meta-analysis of 9 articles to evaluate the association between OSA and the risk of osteoporosis [17]. Their analysis did not find a significant association between osteoporosis and OSA or a difference in bone loss between the groups.

The present study has 4 limitations. First, we did not investigate the OSA of subjects using polysomnography; therefore, the definition of severe snoring in this study was not experimentally verified. However, information about snoring, which can easily be obtained with simple questions, could be useful in pre-screening for fragility fractures. Second, BMD was not measured with DXA. However, qUS has been shown to be a fairly accurate method for measuring BMD [18], and therefore, qUS is being used in the clinical setting along with DXA. Third, fracture history was not obtained from official medical records maintained by physicians but was obtained from survey results, which relied on the memory of the respondents. Moreover, the memory of the respondents about fractures was retrospective up to a 10-year lag, and fracture time was asked by the year. Fourth, this cohort study was designed to investigate the genome-wide association and associated risk factors of chronic disease in urban and rural Koreans; therefore, it might be difficult to extend our findings to the general population. Moreover, there were unmeasured confounders for snoring and fractures, such as mental status, insomnia disorders, dairy product consumption, and pregnancy information.

## Conclusions

This study showed that snoring is significantly associated with fragility fracture risk in women aged 40 years and older. Snoring may be associated with a decrease in BMD and a decrease in the alertness level during the daytime. These 2 health problems are in turn probably associated with an increase in the fracture risk. Information about snoring can easily be obtained with history taking, and treatment options for snoring are available. Thus, information on the frequency of snoring in women may improve the accuracy of fragility fracture risk prediction, which can help in deciding whether intervention or treatment is necessary.

## Additional file

Additional file 1: Table S1. Demographic and clinical characteristics of the male participants in the snoring groups (*n* = 2969). **Table S2.** Crude and adjusted hazard ratios with 95% confidence intervals for fractures among the male participants in Cox regression analyses. (DOC 112 kb)

## Abbreviations

BMD: Bone mineral density
COPD: Chronic obstructive pulmonary disease
KHGS: Korean health and genome study
OSA: Obstructive sleep apnea
qUS: Quantitative ultrasound
SoS: Speed of sound
