# Supplementary material for: Increased fragility fracture risk in Korean women who snore: a 10-year population-based prospective cohort study
Source: BMC Musculoskelet Disord. 2017 May 31;18:236. doi: 10.1186/s12891-017-1587-0 (PMC5452296; doi:10.1186/s12891-017-1587-0)
Supplement: Additional file 1: Table S1. — Demographic and clinical characteristics of the male participants in the snoring groups (n = 2969). Table S2. Crude and adjusted hazard ratios with 95% confidence intervals for fractures among the male participants in Cox regression analyses. (DOC 112 kb) [file 12891_2017_1587_MOESM1_ESM.doc]

**Table S1** Demographic and clinical characteristics of the male participants in the snoring groups (n = 2969)

|  | Non-snoring  (n = 913) | Snoring  (1–5 nights/week)  (n = 1571) | Severe snoring  (6–7 nights/week)  (n = 485) | *p*-value |
| --- | --- | --- | --- | --- |
| Survival time (years) | 9.6 ± 0.9 | 9.6 ± 1.2 | 9.6 ± 0.9 | 0.295 |
| Age (years) | 52.1 ± 9.0 | 50.6 ± 8.2 | 51.1 ± 7.8 | <0.001* |
| Weight (kg) | 65.3 ± 9.1 | 68.7 ± 9.3 | 71.5 ± 9.8 | <0.001* |
| Height (cm) | 166.8 ± 6.0 | 167.2 ± 5.5 | 167.4 ± 5.6 | 0.082 |
| Body mass index (kg/m2) | 23.4 ± 2.7 | 24.5 ± 2.8 | 25.5 ± 3.0 | <0.001* |
| Waist circumference (cm) | 81.5 ± 7.5 | 84.1 ± 7.3 | 86.6 ± 7.5 | <0.001* |
| Hip circumference (cm) | 92.2 ± 5.5 | 93.9 ± 5.6 | 95.7 ± 5.6 | <0.001* |
| Regular exercise (more than 30 min) | 244 (26.7) | 480 (30.6) | 141 (29.1) | 0.129 |
| Alcohol consumption, n (%) |  |  |  | 0.057 |
| Never | 193 (21.1) | 265 (16.9) | 90 (18.6) |  |
| Past | 97 (10.6) | 154 (9.8) | 42 (14.3) |  |
| Current | 623 (68.2) | 1152 (73.3) | 353 (72.8) |  |
| Lifetime smoker, n (%) |  |  |  | 0.531 |
| Never | 200 (21.9) | 304 (19.4) | 93 (19.2) |  |
| Past | 349 (38.2) | 619 (39.4) | 184 (37.9) |  |
| Current | 364 (39.9) | 648 (41.3) | 208 (42.9) |  |
| History of previous fracture, n (%) | 21 (2.3) | 59 (3.8) | 21 (4.3) | 0.073 |
| Family history of osteoporosis or fracture, n (%) | 34 (3.7) | 77 (4.9) | 31 (6.4) | 0.080 |
| Degenerative arthritis, n (%) | 36 (3.9) | 86 (5.5) | 29 (6.0) | 0.152 |
| Rheumatoid arthritis, n (%) | 15 (1.6) | 36 (2.3) | 6 (1.2) | 0.256 |
| Hypertension, n (%) | 85 (9.3) | 234 (14.9) | 83 (17.1) | <0.001* |
| Diabetes mellitus, n (%) | 67 (7.3) | 112 (7.1) | 35 (7.2) | 0.981 |
| Medications for osteoporosis, n (%) | 0 (0.0) | 1 (0.1) | 1 (0.2) | 0.367 |
| Speed of sound of radius (m/s) | 4158.2 ± 147.9 | 4149.0 ± 148.6 | 4132.2 ± 147.0 | 0.008* |
| T-score of radius | 0.3 ± 1.2 | 0.2 ± 1.2 | 0.1 ± 1.2 | 0.007* |
| Z-score of radius | 0.6 ± 1.2 | 0.5 ± 1.2 | 0.4 ± 1.2 | 0.002* |
| Speed of sound of tibia (m/s) | 3985.5 ± 121.6 | 3984.7 ± 114.9 | 3980.5 ± 114.6 | 0.729 |
| T-score of tibia | 0.3 ± 1.2 | 0.3 ± 1.1 | 0.2 ± 1.1 | 0.634 |
| Z-score of tibia | 0.7 ± 1.2 | 0.7 ± 1.2 | 0.7 ± 1.2 | 0.538 |
| Fracture, n (%) | 32 (3.5) | 76 (4.8) | 21 (4.3) | 0.291 |

Data are presented as mean ± standard deviation or number of participants (%).

The *p*-values of continuous and binary variables were calculated using analysis of variance (ANOVA) and the chi-square test, respectively.

**p* < 0.05

**Table S2** Crude and adjusted hazard ratios with 95% confidence intervals for fractures among the male participants in Cox regression analyses

|  | Fracture | | Model 1 | | Model 2 | | Model 3 | |
| --- | --- | --- | --- | --- | --- | --- | --- | --- |
|  | No (n = 2840) | Yes (n = 129) | *p*-value | HR (95% CI) | *p*-value | HR (95% CI) | *p*-value | HR (95% CI) |
| Survival time (years) | 9.7 ± 0.7 | 6.6 ± 2.5 |  |  |  |  |  |  |
| Snoring, n (%) |  |  |  |  |  |  |  |  |
| Non-snoring | 881 (31.0%) | 32 (24.8%) |  |  |  |  |  |  |
| Snoring | 1465 (52.6%) | 76 (58.9%) | 0.120 | 1.388 (0.918–2.098) | 0.136 | 1.378 (0.904–2.099) |  |  |
| Severe snoring | 464 (16.3%) | 21 (16.3%) | 0.457 | 1.232 (0.711–2.137) | 0.616 | 1.158 (0.652–2.057) |  |  |
| Age (years) | 51.2 ± 8.4 | 51.2 ± 8.6 |  |  | 0.620 | 1.006 (0.982–1.031) |  |  |
| Weight (kg) | 68.1 ± 9.6 | 69.0 ± 9.4 |  |  | 0.349 | 0.976 (0.927–1.027) |  |  |
| Height (cm) | 167.1 ± 5.7 | 168.1 ± 5.4 |  |  | 0.009* | 1.057 (1.014–1.102) | 0.016* | 1.038 (1.007–1.071) |
| Body mass index (kg/m^2^) | 24.3 ± 2.9 | 24.4 ± 2.8 |  |  |  |  |  |  |
| Waist circumference (cm) | 83.7 ± 7.6 | 84.4 ± 7.9 |  |  | 0.408 | 1.020 (0.973–1.069) |  |  |
| Hip circumference (cm) | 93.7 ± 5.7 | 94.1 ± 6.0 |  |  | 0.814 | 1.007 (0.951–1.067) |  |  |
| Regular exercise (more than 30 min) | 831 (29.3%) | 34 (26.4%) |  |  | 0.482 | 0.866 (0.579–1.294) |  |  |
| Alcohol consumption, n (%) |  |  |  |  |  |  |  |  |
| Never | 528 (18.6%) | 20 (15.5%) |  |  | Reference | |  |  |
| Former | 280 (9.9%) | 13 (10.1%) |  |  | 0.626 | 1.193 (0.586–2.427) |  |  |
| Current | 2032 (71.6%) | 96 (74.4%) |  |  | 0.542 | 1.165 (0.690–1.954) |  |  |
| Lifetime smoker, n (%) |  |  |  |  |  |  |  |  |
| Never | 576 (20.3%) | 21 (16.3%) |  |  | Reference | |  |  |
| Former | 1104 (38.9%) | 48 (37.2%) |  |  | 0.574 | 1.161 (0.690–1.954) |  |  |
| Current | 1160 (40.9%) | 60 (46.5%) |  |  | 0.222 | 1.376 (0.824–2.298) |  |  |
| History of previous fracture, n (%) | 97 (3.4%) | 4 (3.1%) |  |  | 0.708 | 0.825 (0.302–2.255) |  |  |
| Family history of osteoporosis or fracture, n (%) | 135 (4.8%) | 7 (5.4%) |  |  | 0.769 | 1.122 (0.521–2.417) |  |  |
| Degenerative arthritis, n (%) | 142 (5.0%) | 9 (7.0%) |  |  | 0.515 | 1.277 (0.611–2.668) |  |  |
| Rheumatoid arthritis, n (%) | 52 (1.8%) | 5 (3.9%) |  |  | 0.181 | 1.919 (0.738–4.988) | 0.079 | 2.231 (0.911-5.462) |
| Hypertension, n (%) | 387 (13.6%) | 15 (11.6%) |  |  | 0.462 | 0.810 (0.463–1.419) | 0.001* | 31.487 (4.319–229.558) |
| Diabetes mellitus, n (%) | 207 (7.3%) | 7 (5.4%) |  |  | 0.306 | 0.655 (0.291–1.473) |  |  |
| Medications for osteoporosis, n (%) | 1 (0.0%) | 1 (0.8%) |  |  | <0.001* | 48.867 (5.567–428.972) |  |  |
| Speed of sound in radius (m/s) | 4149.7 ± 148.1 | 4136.0 ± 152.4 |  |  | 0.500 | 1.000 (0.998–1.001) |  |  |
| Speed of sound in tibia (m/s) | 3985.3 ± 117.0 | 3962.0 ± 113.1 |  |  | 0.017* | 0.998 (0.997–1.000) | 0.010* | 0.998 (0.997–1.000) |

HR = hazard ratio; CI = confidence interval

Data are presented as mean ± standard deviation or number of participants (%).

Crude univariate Cox regression model 1 did not control for any covariate.

Cox regression model 2 controlled for age, weight, height, waist circumference, hip circumference, regular exercise, alcohol consumption, lifetime smoker, history of fracture, family history of osteoporosis or fracture, degenerative arthritis, rheumatoid arthritis, hypertension, diabetes mellitus, and quantitative ultrasound measurements at the radius and tibia.

Cox regression model 3 with backward stepwise elimination involved a threshold of *p* = 0.1.

**p* < 0.05
